# Hereditary Neuropathy with Liability to Pressure Palsies

**DOI:** 10.15844/pedneurbriefs-29-11-2

**Published:** 2015-11

**Authors:** Hyoung Won Choi, Nancy L. Kuntz

**Affiliations:** 1 Division of Neurology, Ann & Robert H. Lurie Children's Hospital of Chicago, Chicago, IL; 2 Departments of Pediatrics and Neurology, Northwestern University Feinberg School of Medicine, Chicago, IL

**Keywords:** Mononeuropathies, HNPP, Childhood, PMP22 Protein

## Abstract

Investigators from 4 pediatric hospitals in Canada analyzed the clinical presentation and electrophysiological data of 12 children with hereditary neuropathy with liability to pressure palsies (HNPP), caused by PMP22 gene deletion.

Investigators from 4 pediatric hospitals in Canada analyzed the clinical presentation and electrophysiological data of 12 children with hereditary neuropathy with liability to pressure palsies (HNPP), caused by PMP22 gene deletion. Peroneal palsy was the most common presentation (42%) followed by brachial plexus palsy in 25% of their cases. Complete nerve conduction studies were available in 10/12 cases and it demonstrated 3 major patterns: multifocal demyelination at areas of nerve entrapment without generalized demyelinating polyneuropathy (20%), isolated generalized sensorimotor polyneuropathy (20%), combined focal demyelination at the area of entrapment and demyelinating polyneuropathy (60%). All patients had electrophysiological evidence of unilateral or bilateral carpal tunnel syndrome, although it was not always symptomatic. Electrophysiological findings are useful in diagnosis of HNPP, especially in children with heterogeneous clinical presentation. [1]

COMMENTARY. HNPP is the third most common type of hereditary motor and sensory neuropathy [1]. The typical clinical presentation is usually reported as a recurrent, painless monoparesis, with its first attack being in second or third decades [2]. The diagnosis is usually made in early adulthood unless there is a family history. A smaller case series published by Potulska-Chromik et al. about 7 children with genetically confirmed HNPP provides similar observations [3]. Their clinical presentation varied from mononeuropathy to brachial plexopathy, with recurrent episodes in 4 out of 7 patients. An earlier study that included 48 children with HNPP documented > 50% of children with HNPP has delayed onset of walking (after 15 months of age) [4]. Based on the results of the study, the authors advocate testing PMP22 gene deletion for any children with unexplained mononeuropathy or multifocal neuropathy to facilitate appropriate care and genetic counseling for these patients. The paper alerts child neurologists to consider the possibility of HNPP even in young children with a negative family history when they present with the typical compressive nerve palsy.
